# The effects of platelet-rich plasma injections in different stages of knee osteoarthritis

**DOI:** 10.1007/s00590-023-03481-6

**Published:** 2023-02-02

**Authors:** Juho A Annaniemi, Jüri Pere, Salvatore Giordano

**Affiliations:** 1 Department of Surgery, Welfare District of Forssa, Forssa, Finland; 2 Department of Orthopaedics and Traumatology, Turku University Hospital, Kiinamyllynkatu 4-8, 20521 Turku, Finland; 3 Department of General and Plastic Surgery, Turku University Hospital and University of Turku, Turku, Finland; 4 University of Turku, Turku, Finland

**Keywords:** Platelet-rich plasma, Injections, Knee, Osteoarthritis

## Abstract

**Purpose:**

Platelet-rich plasma (PRP) injections are challenging the current treatment options for knee osteoarthritis (KOA). Targeting the treatment to optimal stage of the symptomatic KOA may be crucial in the success and failure of treatment. The aim of this study is to compare the outcomes of PRP injections at different stages of KOA in order to determine the optimal stage of the KOA for PRP injection treatments.

**Methods:**

A total of 89 consecutive patients with symptomatic KOA Kellgren–Lawrence grade 1 to 3 (Group A grade 1, group B grade 2 and group C grade 3) were given three intra-articular injections of PRP with 2 weeks interval between injections. Visual Analogue Scale (VAS), Western Ontario and McMaster Universities Osteoarthritis Index (WOMAC) and Range of Motion were measured before injection, at 15 days, 6 months, 12 months and, at last follow-up. Intergroup outcomes were compared.

**Results:**

The comparison of groups A and C showed that WOMAC scores were significantly higher in group C at 15 days [*p* = 0.047] and at last follow-up [*p* = 0.008] than in group A, as well as VAS scores at 6 months [*p* = 0.031] and at last follow-up [*p* = 0.008]. The overall WOMAC and VAS scores showed decrease in all the groups in minimum follow-up of 14 months. The other comparisons did not show significant differences in outcomes.

**Conclusion:**

All the groups showed decrease in WOMAC and VAS, but patients with mild KOA benefit significantly more from the treatments than patients with more severe KOA.

## Introduction

Knee osteoarthritis (KOA) affects millions of people globally and is currently leading cause of disability in elderly people. [1, 2] Treatment options for KOA include exercise programs, weight management, pain medication, intra-articular injections and, arthroplasty surgery, which is usually reserved as the last line treatment. [3, 4] The role of platelet-rich plasma (PRP) injections is still under debate and currently America Academy of Orthopaedic Surgeons (AAOS) recommendation for the use of PRP in KOA is limited but not excluded from clinical practise. [5]

PRP injections have showed to reduce clinical symptoms such as pain and improved physical outcome scores in previous studies. [6–10] Subjective reported duration of the PRP treatment is approximately 12 months and need for reintervention for PRP treated patients is lower when compared to intra-articular hyaluronic acid injections. [7] Several studies have reported that PRP is superior to placebo and even to other forms of intra-articular injection therapies. [6, 8, 9] PRP treatments also seem to delay the need for arthroplasty. [11, 12]

Despite rigorous studies, uncertainties remain, especially regarding patient selection in PRP treatments. To clarify the patient choice, the effectiveness of PRP injections should be compared in different stages of KOA. This would provide clinically meaningful information to support optimal treatment selection for patients suffering from KOA. Osteoarthritis (OA) is radiographically evaluated with Kellgren–Lawrence (KL) grading from 0 to 4 scale, grade 0 being definite absence of X-ray changes in OA and grade 4 being severe changes in OA. [13] Considering PRP treatments, only few studies compare the efficacy of PRP in different stages of KOA, with short term and poor results with KL grade 4 OA but with better results in mild to moderate OA. [14, 15]

The goal of our study is to determine if there is any significant difference in the clinical outcomes of the intra-articular PRP injections at different stages of KOA. We hypothesized that patients with earlier stages of KOA are likely to benefit more from PRP treatment than patients with later stages. If there are differences in PRP efficacy between the milder forms of KOA, this would be meaningful information in clinical decision-making when treating milder forms of KOA. The results would also sharpen the indications of intra-articular PRP injections when treating KOA patients.

## Materials and methods

This is a retrospective study including a total of 89 consecutive patients with symptomatic KOA who received PRP-injections. Patients had received intra-articular PRP injections to their knee between January 2014 and October 2017 at the Welfare District of Forssa, Finland. Research data were collected from electronic medical records. This study followed ethical principles of the World Medical Association Declaration of Helsinki, and the study was approved by the Institutional Review Board. Individual informed consent was waived due to de-identification of source data.

Inclusion criteria were the following: KL grade 1 to 3 knee OA in radiographic imaging, pre-intervention pain Visual Analogue Scale (VAS) of 30 to 100 and age between 18 and 90 years. Exclusion criteria were: Age below 18 or above 90, major symptomatic hip OA of the same side, major systemic diseases (hematological diseases, infections, immunodeficiency, active of fulminant rheumatoid disease), pregnancy or possibility of pregnancy.

Demographic data were meticulously collected from the electronic medical records, as well as the preintervention and follow-up data. Follow-up parameters included Visual Analogue Scale (VAS), Western Ontario and McMaster Universities Osteoarthritis Index (WOMAC) and Range of Motion (ROM) of the knee, which were documented before injections, at 15 days after first injection, at 6 months, at 12 months and/or at the last follow-up. Patients were allocated to three study arms for the analysis according to preintervention Kellgren–Lawrence grading of the preintervention radiographs (Group A grade 1, group B grade 2 and group C grade 3) for the purpose of this study.

The PRP injections were performed using the commercial Glo PRP kit (GloFinn corporation, Salo, Finland), which resulted in autologous PRP manufactured from patients’ own whole blood. Patients’ blood was drawn up to 9 to 10 ml in the kit’s syringe, and it was centrifuged for 5 min at 1200 rpm. The excess red blood cells were removed, and the product was centrifuged again for 10 min at 1200 rpm. Final product reached approximately 4 to 8 times higher concentration of platelets compared to whole blood. One treatment cycle consisted of 3 intra-articular injections, with single injection containing approximately 5 ml of PRP. White blood cells were not separated from the final product. Injections were given at 10 to 14 days of interval. The injection was performed by an experienced orthopaedist by using anatomical landmarks and aspiration to reach the target intra-articular space of the knee.

Continuous variables were reported as the mean ± standard deviation. Normality assumptions were demonstrated with histograms, Skewness, Kurtosis, and Kolmogorov/Smirnov tests. Pearson’s chi-square test, Fisher’s exact test, and the Mann–Whitney test or *t *test were used for univariate analysis, as appropriate, for comparisons between the study groups according to the Kellgren–Lawrence grading.

A two-sided *p *value less than 0.05 was considered statistically significant. All analyses were carried out using SPSS statistical software (IBM SPSS Statistics, version 23, Armonk, NY).

## Results

**Fig. 1:**
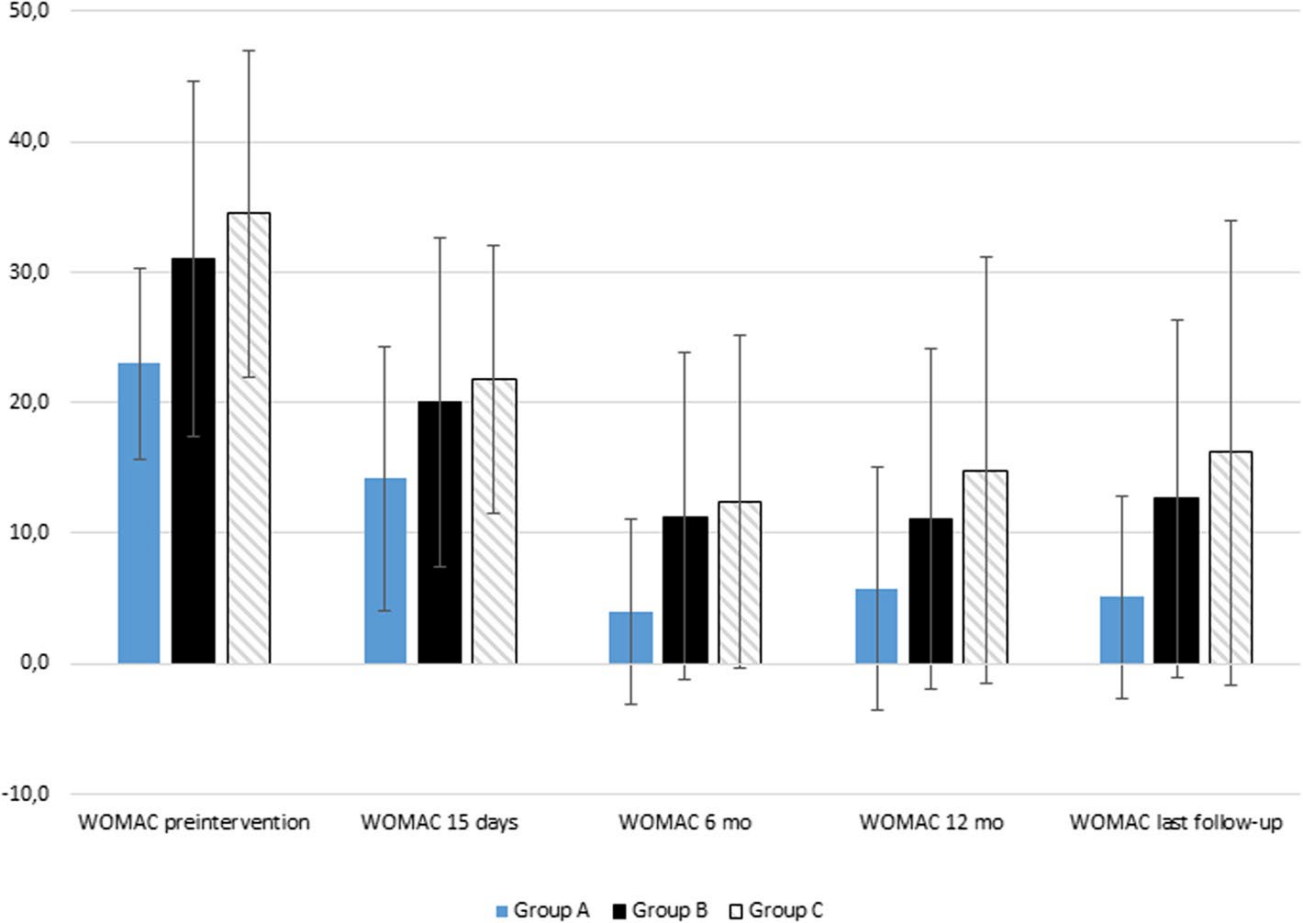
Mean WOMAC scores of the patients during the follow-up with ± 1 standard deviation

The demographic and pre-interventional data were similar in each of the group from A to C, with mean follow-up of at least 14 months in each group, apart from the WOMAC overall score, which was significantly higher in the group C [*p* = 0.013] than in group A, but no difference between groups B and C was detected. [Table 1, Fig. 1] No differences in obesity or comorbidities were noticed between any the study groups.

**Table 1:** Baseline characteristics of patients who underwent intra-articular injections of PRP for different stages of knee Osteoarthritis (OA) grade according to Kellgren–Lawrence classification (1–3)

	Group A (OA1)	Group B (OA2)	Group C (OA3)	*P *value^*a*^	*P *value^*b*^	*P *value^*c*^
	*n* = 9	*n* = 49	*n* = 33			
Mean age (years)	59.9 ± 10.0	56.6 ± 10.4	58.1 ± 10.4	0.390	0.536	0.646
Females	6 (66.7%)	30 (61.2%)	20 (60.6%)	1.000	0.955	0.724
Mean BMI^†^ (kg/m^2^)	27.4 ± 5.2	28.9 ± 4.1	29.1 ± 5.2	0.356	0.808	0.391
Obese (BMI > 30 kg/m^2^)	2 (22.2%)	17 (34.7%)	14 (42.4%)	0.703	0.479	0.283
Comorbidity	3 (33.3%)	16 (32.7%)	13 (39.4%)	1.000	0.531	0.724
Diabetes	2 (22.2%)	4 (8.2%)	1 (3.0%)	0.231	0.408	0.116
Cardiac disease	1 (11.1%)	7 (14.3%)	2 (6.1%)	1.000	0.302	1.000
Smokers	1 (11.1%)	9 (18.4%)	5 (15.2%)	0.512	0.704	1.000
Bilateral	0	9 (18.4%)	4 (12.1%)	0.328	0.547	0.559
Flexion degree	135.0 ± 12.2	132.5 ± 20.3	127.0 ± 18.1	0.726	0.207	0.218
Extension degree	88.3 ± 3.5	88.8 ± 3.1	88.3 ± 3.0	0.705	0.526	1.000
VAS pain score (0–100)	58.3 ± 15.4	66.6 ± 14.8	65.0 ± 21.4	0.131	0.678	0.391
WOMAC overall	23.0 ± 7.3	31.0 ± 13.6	34.5 ± 12.5	0.093	0.247	***0.013***
Follow-up (months)	14.8 ± 4.9	14.0 ± 4.4	15.0 ± 6.5	0.638	0.409	0.924

*P* < 0.05 values were considered statistically significant and are bolded in the table

^†^ BMI; body mass index

^a^Group A versus group B

^b^Group B versus group C

^c^Group A versus group C

**Fig. 2:**
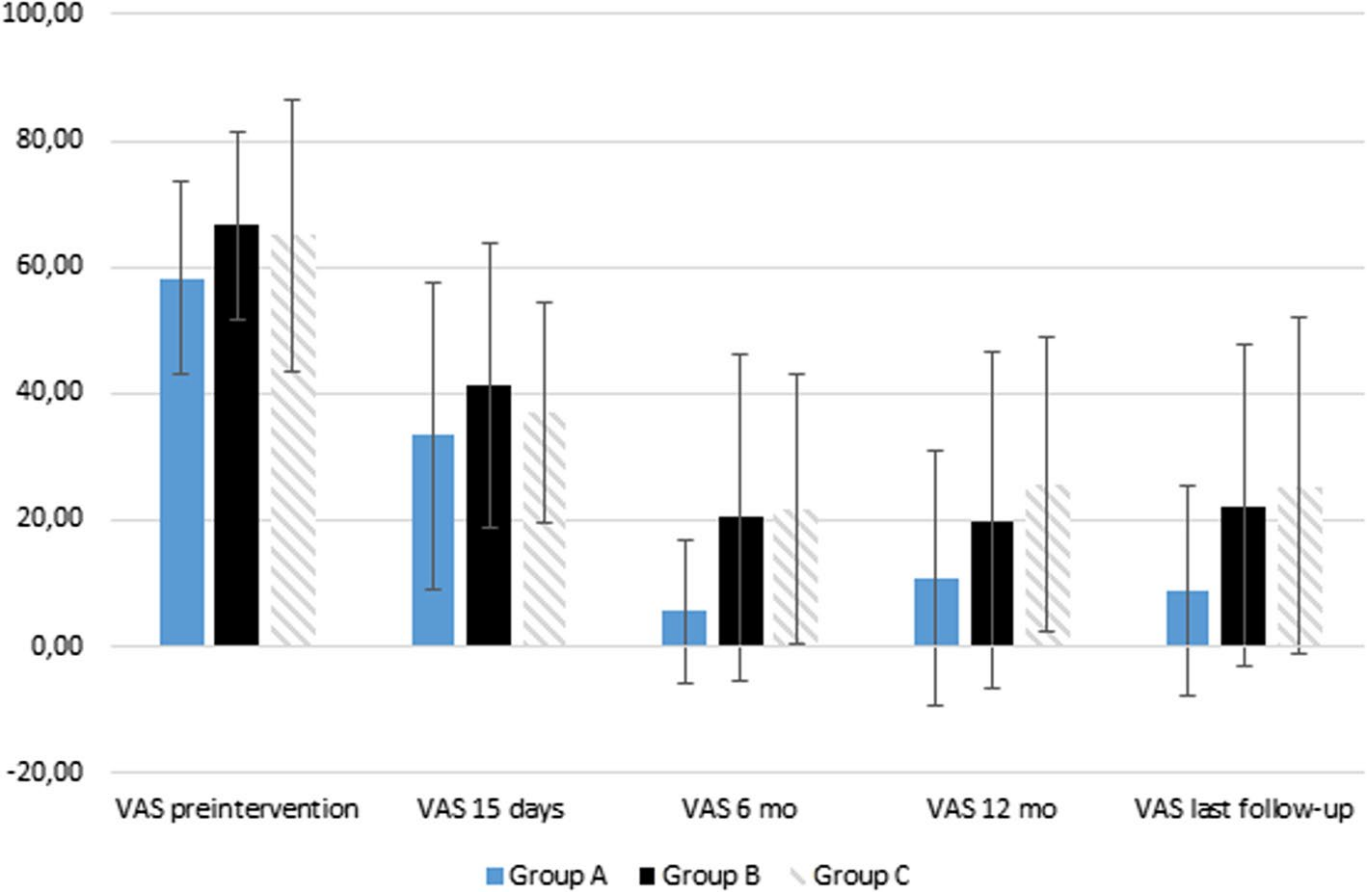
Mean VAS scores of the patients during the follow-up with ± 1 standard deviation

We did not find statistically differences between groups A and B at any point of the follow-up or in the pre-intervention parameters. [Tables 1, 2] During the follow-up, group C showed poorer results in WOMAC, VAS and ROM than other groups. [Table 2] Especially WOMAC scores were lower in group A than in group C at 15 days [14.2 ± 10.1 vs. 21.8 ± 10.3, *p* = 0.047] and last follow-up [5.1 ± 7.8 vs. 16.2 ± 17.8, *p* = 0.008]. [Table 2] VAS scores also showed statistically significant differences between group A and group C, with again poorer results in group C at 6 months [5.6 ± 11.3 vs. 21.6 ± 21.4, *p* = 0.031] and last follow-up [8.9 ± 16.7 vs. 25.3 ± 26.6, *p* = 0.029]. [Table 2, Fig. 2].

**Table 2:** Outcomes of patients who underwent intra-articular injections of PRP for different stages of knee Osteoarthritis (OA) grade according to Kellgren–Lawrence classification (1–3)

	Group A (KL1)	Group B (KL2)	Group C (KL3)	*P* value^*a*^	*P* value^*b*^	*P* value^*c*^
	*n* = 9	*n* = 49	*n* = 33			
Adverse events	0	1 (2.0%)	3 (9.1%)	1.000	0.297	0.581
Number of injections	3.0 ± 0	3.1 ± 0.6	3.5 ± 1.8	0.706	0.151	0.418
Flexion degree (15 days)	137.22 ± 12.5	136.33 ± 17.0	130.4 ± 15.3	0.881	0.115	0.217
Extension degree (15 days)	88.9 ± 2.2	89.3 ± 2.3	88.9 ± 2.4	0.632	0.513	0.985
VAS pain score (0–100)—(15 days)	33.3 ± 24.4	41.2 ± 22.5	37.0 ± 17.4	0.343	0.362	0.628
WOMAC overall (15 days)	14.2 ± 10.1	20.1 ± 12.6	21.8 ± 10.3	0.192	0.518	***0.047***
Flexion degree (6 months)	141.7 ± 9.3	139.3 ± 16.2	134.2 ± 15.4	0.672	0.166	0.175
Extension degree (6 months)	90.0 ± 0	90.3 ± 4.6	89.4 ± 1.7	0.844	0.277	0.267
VAS pain score (0–100)—(6 months)	5.6 ± 11.3	20.4 ± 25.8	21.6 ± 21.4	0.096	0.840	***0.031***
WOMAC overall (6 months)	4.0 ± 7.1	11.3 ± 12.5	12.4 ± 12.7	0.099	0.684	*0.054*
Flexion degree (12 months)	139.2 ± 10.2	139.8 ± 14.6	134.3 ± 15.6	0.925	0.158	0.475
Extension degree (12 months)	90.0 ± 0	89.5 ± 1.9	88.7 ± 2.6	0.530	0.143	0.232
VAS pain score (0–100)—(12 months)	10.8 ± 20.1	19.9 ± 26.5	25.6 ± 23.3	0.426	0.373	0.138
WOMAC overall (12 months)	5.8 ± 9.3	11.1 ± 13.0	14.8 ± 16.3	0.347	0.302	0.181
Flexion degree (last follow-up)	140.6 ± 8.8	139.5 ± 15.0	134.4 ± 16.6	0.838	0.153	0.287
Extension degree (last follow-up)	90.0 ± 0	89.5 ± 1.8	88.5 ± 2.9	0.412	0.060	***0.005***
VAS pain score (0–100)—(last follow-up)	8.9 ± 16.7	22.3 ± 25.4	25.3 ± 26.6	0.135	0.608	***0.029***
WOMAC overall last follow-up)	5.1 ± 7.8	12.7 ± 13.7	16.2 ± 17.8	0.113	0.311	***0.008***
Any knee arthroplasty	0	1 (2.0%)	4 (12.1%)	1.000	0.152	0.561
Unicompartmental knee arthroplasty	0	0	2 (6.0%)	–	0.159	1.000
Total knee arthroplasty	0	1 (2.0%)	2 (6.0%)	1.000	0.562	1.000

*P* < 0.05 values were considered statistically significant and are bolded in the table

^a^Group A versus group B

^b^Group B versus group C

^c^Group A versus group C

In addition, there was a difference in ROM extension degree at last follow-up favouring groups A over group C [90.0 ± 0 vs. 88.5 ± 2.9, *p* = 0.005]. [Table 2] Four patients from group C ended up in arthroplasty, one patient from group B and none from group A. [Table 2] Adverse events were also documented, and group A had none, group B had a single adverse event and group C had 3 adverse events from the injections. [Table 2] The adverse event in group B was prolonged pain for one week after the second injection, and the three adverse events in group C were prolonged pain for one week after second and third injection.

## Discussion

This study sought to find a potential subgroup that would benefit more from intra-articular PRP injections in a population with Kellgren–Lawrence grade 1 to 3 KOA. Although, all the groups showed overall decrease in WOMAC scores during the follow-up, the analysis revealed that patients with very mild KOA benefit significantly more from this treatment modality than patients with more severe graded OA. Therefore, patients with KL 1 KOA may have a slight edge over KL 2 and 3 patients in terms of PRP efficacy outcomes. The mean VAS and WOMAC scores of all the groups plummet during the first 6 to 12 months, and there still is finite but significant difference between the more severe and less severe KOA in VAS scores at 6 months. WOMAC scores also come close to being statistically significant at 6 months.

The differences in VAS and WOMAC scores between group A and C probably adhere to the severity of the KOA. Previous studies indicate that the process of OA involves complex interplay of several cytokines, matrix metalloproteinases and mechanical stress, which create a vicious cycle that drives the cartilage towards degeneration and destruction through inflammation and apoptosis. [16–18] In contrast, the effects of PRP are believed to affect these molecular mechanisms in the cartilage and surrounding tissues by diminishing the inflammation and balancing the homeostasis of the joint. [19] The joint becomes more and more destroyed as the OA progresses inevitably, which probably makes it more difficult for PRP to have effect in the joint.

Surprisingly, we also detected a significant difference in ROM extension at the last follow-up point. This is probably due to some of the symptoms returning faster in group C than in group A or B after time has passed, thus, the difference is probably due to different speed of symptoms returning in each group. The ROM extension, VAS and WOMAC data suggest that group A may have longest benefit from the PRP injections because their mean scores tend to stay rather low, and in turn groups B and C experience slight increase in their mean scores.

Adverse events documented in this study were prolonged post injection pain in the knee and mild effusion of the joint. Prolonged effusion and pain resolved after 5 days. No serious adverse events were detected. The adverse events documented were all prolonged pain after second and third injection which resolved in 1 week. This is probably inherent to injection therapy in general. A total of four patients from group C (12.1%) and one patient from group B (2.0%) underwent knee arthroplasty. None of the patients in group A had any arthroplasties.

The strengths of this study include moderate follow-up length, meticulously collected data of the symptom scores, demographic data, and adequate sample size. Adverse events and patients who underwent knee arthroplasties were also documented.

The limitations of this study include smaller number of patients in group A, sex-ratio leaning towards females over males in each group and pre-intervention difference in WOMAC overall score between the groups A and C. Also, the retrospective nature of this study is an important limitation, although the patients were consecutive. We did not include KL grade 4 patients in this study because the previous evidence does not support the use of PRP in KL 4 KOA, and thus, the comparison was unnecessary. [14] The small number of patients in group A is likely to be a natural selection bias as the mildest stage of KOA is unlikely to cause enough symptoms for most of the patients that would make them actively seek medical help, therefore, finding patients with enough symptoms with KL 1 KOA is difficult. PRP preparations and injections protocol might differ from other studies, but we followed the Glo PRP kit manufacturer’s instructions (GloFinn corporation, Salo, Finland).

The clinical significance of this study is that patients might receive help from PRP injections from KL grade 1 to 3 KOA and the outcomes are significantly better with lower KL grade of the OA. This might be a useful information when choosing the treatment modality for symptomatic KOA, providing optimal long-lasting improvement for patients’ symptoms or considering prolonging the arthroplasty surgery. It is also important to have other viable options besides acetaminophen (APAP) and non-steroidal anti-inflammatory drugs (NSAID), that are beneficial until the inevitable total loss of cartilage on the joint surface. Viable and safe alternative options are especially called for elderly patients, whom may have diseases or medications that are contraindication for NSAID or APAP usage. Also, younger patients may prefer injections over oral or topical medication, especially if the injection provides relatively long symptoms’ alleviation. Therefore, this study may contribute to the clinical decision-making when treating patients with KOA that are not yet ripe for arthroplasty.

In the light of our results, PRP seems to be adequate treatment for KL 1 to 3 KOA. All the groups experienced drastic reduction in their symptoms. Group A with the mildest KL 1 KOA had a small but statistically significant edge over the other groups. Adverse events were rare, and effects of the adverse events were minimal, and probably inherent to intra-articular injections in general. Only few patients underwent knee arthroplasty during the follow-up of over a year, which is a positive indication of the treatment working beneficially enough for the patients even with KL 3 KOA. The results would suggest that optimal treatment window for PRP covers the entire spectrum of KOA, all the way until the joint is ripe for arthroplasty. As a conclusion, intra-articular injections of PRP have a diminishing effect as the KOA progresses, however, meaningful effects are seen throughout KL 1 to 3 grades, but KL 1 graded KOA patients seem to receive most help from the treatment.
